# Language Barriers in the Federal Republic of Somalia's Healthcare: Addressing Inclusivity for Minority Dialects

**DOI:** 10.1002/hcs2.70043

**Published:** 2025-12-07

**Authors:** Najib Isse Dirie, Mohamed Mustaf Ahmed

**Affiliations:** ^1^ Department of Urology, Dr. Sumait Hospital, Faculty of Medicine and Health Sciences SIMAD University Mogadishu The Federal Republic of Somalia; ^2^ SIMAD Institute for Global Health, Faculty of Medicine and Health Sciences SIMAD University Mogadishu The Federal Republic of Somalia

**Keywords:** digital health, healthcare inclusivity, language barriers, minority dialects, The Federal Republic of Somalia, universal health coverage

## 1 Introduction

The Federal Republic of Somalia, often perceived as linguistically homogeneous, is home to a rich tapestry of dialects and minority languages that reflect its diverse cultural heritage. While Somali is the official medium of communication, it is divided into two major dialects: Maxaa Tiri (spoken by approximately 60% of the population) and Maay (spoken by approximately 20% of the population) [1]. Minority languages such as Bravanese (also known as Chimwiini or Chimbalazi), Mushunguli, Benadiri Somali, and Kibajuni are spoken by smaller communities, particularly in the southern and coastal regions [1]. Despite the demographic linguistic diversity shown in Table 1, Maxaa Tiri holds institutional dominance, functioning as the primary dialect in healthcare delivery, official communication, and in documentation [2]. This dominance marginalizes Maay speakers and other minority languages, limiting their access to healthcare information and services [2]. Effective communication between healthcare providers and patients is essential for informed consent, accurate diagnosis, appropriate treatment, and patient safety [3]. However, language barriers prevent many minority‑language speakers from accessing these critical aspects of care.

**Table 1:** Distribution of dominant and minority languages across Somali regions. Source: Translators without Borders, 2021 [1].

Region	Dominant language	Dominant language share (%)	Benadiri Somali	Bravanese Swahili	Bajuni Swahili	Maay Somali	Mushungulu	Somali	Total population
Awdal	Somali	97	0.006	0	0	0	0	0.97	724,571
Bakool	Maay Somali	88	0.036	0.002	0.006	0.88	0.005	0.045	284,355
Banadir	Benadiri Somali	49	0.493	0	0	0.262	0.001	0.226	2,228,463
Bari	Somali	83	0.101	0	0	0.041	0	0.836	712,931
Bay	Maay Somali	96	0.004	0	0	0.961	0	0.031	846,600
Galgaduud	Somali	83	0.147	0	0	0.01	0	0.831	427,810
Gedo	Somali	84	0.026	0	0	0.126	0	0.848	430,940
Hiraan	Somali	51	0.398	0	0	0.076	0.001	0.513	422,991
Lower Juba	Somali	84	0.058	0	0.007	0.082	0.007	0.846	648,939
Lower Shabelle	Maay Somali	44	0.407	0	0	0.44	0.004	0.149	911,499
Middle Shabelle	Benadiri Somali	47	0.479	0	0	0.163	0.022	0.331	436,760
Mudug	Somali	92	0.044	0	0	0.03	0.001	0.92	627,721
Nugaal	Somali	95	0.031	0	0	0.008	0	0.955	337,590
Sanaag	Somali	99	0	0	0	0	0	0.999	562,066
Sool	Somali	98	0.003	0	0.002	0	0	0.989	360,432
Togdheer	Somali	99	0.003	0	0	0	0	0.997	755,794
Woqooyi Galbeed	Somali	100	0	0	0	0	0	1	1,321,524
Middle Juba	—								2,86,540

Health campaigns and medical documentation are predominantly conducted in Maxaa Tiri, leaving minority language speakers unable to fully understand vital health information [2, 4]. Rural areas in the Federal Republic of Somalia are particularly vulnerable to the consequences of this linguistic exclusion. These regions often lack sufficient healthcare infrastructure and are home to many speakers of Maay and other minority dialects [2, 4]. In such areas, healthcare workers may not speak local dialects, leading to frequent miscommunication. Moreover, health information, whether disseminated via campaigns, posters, or mobile platforms, is almost exclusively provided in Maxaa Tiri. This renders it inaccessible to large rural populations in developing countries. Consequently, residents of these regions are less likely to engage with health services, contributing to lower vaccination rates, delays in seeking care and poorer health outcomes.

This disconnection likely contributes to poorer health outcomes in minority communities. Although specific statistical data quantifying these outcomes are currently unavailable for the Federal Republic of Somalia, studies from similar low‐resource settings have consistently demonstrated that language barriers lead to decreased healthcare utilization, reduced adherence to medical instructions, and increased patient safety risk [5, 6]. This commentary analyzes the linguistic barriers faced by minority language speakers in accessing healthcare in the Federal Republic of Somalia. It explores solutions through inclusive policies, digital health platforms and global best practices. By addressing these challenges, the Federal Republic of Somalia can move closer to achieving equitable healthcare for all its citizens and meet the Universal Health Coverage (UHC) goals [7]. To better understand the nature of these challenges, it is important to first examine the Federal Republic of Somalia's complex linguistic landscape and its interaction with institutional healthcare practices. As outlined in Table 1, the Federal Republic of Somalia exhibits a rich tapestry of linguistic diversity, with dialects such as Maxaa Tiri, Maay, and various minority languages spoken across the regions. However, healthcare institutions primarily favor Maxaa Tiri in their official practices [4]. This linguistic exclusivity leaves minority language speakers struggling to communicate with providers or understand medical advice [4]. The distribution of minority languages, particularly in the southern and coastal regions, highlights the complexity of providing equitable healthcare across linguistically diverse areas. Patients from these communities often avoid seeking care due to fear of miscommunication or mistreatment [4]. This exclusion contributes to poorer health outcomes for marginalized groups and widens the inequities within the Federal Republic of Somalia's healthcare system. Language barriers significantly hinder informed consent processes and effective communication during medical treatment in the Federal Republic of Somalia's health care system [8]. Informed consent requires that patients fully understand the nature, risks, and benefits of medical procedures before giving approval [9]. However, most consent forms are prepared exclusively in Maxaa Tiri, leaving minority language speakers unable to fully comprehend their content [4].

This lack of comprehension undermines ethical principles such as autonomy and exposes both patients and providers to potential risks. For example, patients may unknowingly agree with procedures without understanding the potential complications or alternative treatments due to linguistic barriers [10]. The significance of addressing these barriers was underscored during 2024 UHC Day in the Federal Republic of Somalia, as highlighted by the United Nations. The event emphasized the need for inclusive healthcare systems that cater to diverse linguistic communities. Innovative approaches, such as using theater and visual imagery, have been shown to be effective tools for raising awareness of health issues among populations with limited literacy or language proficiency [11]. These methods can bridge communication gaps by delivering critical health messages in an accessible and engaging manner. Communication challenges extend beyond consent forms to interactions during the treatment. Patients who cannot effectively describe symptoms or understand the instructions from providers are at risk of misdiagnosis or improper care [12]. These issues are particularly acute during emergencies when timely communication is critical. To address these challenges, it is essential to employ trained interpreters or bilingual staff in healthcare facilities. Visual aids or pictorial consent forms could also help low‐literacy populations understand critical information more effectively.

Digital health platforms have emerged as promising tools for addressing healthcare challenges in the Federal Republic of Somalia, particularly in regions with limited physical infrastructure [13]. Platforms such as Baano and SomDoctor have introduced telehealth services that allow patients to consult providers remotely [13, 14]. However, these platforms primarily cater to Maxaa Tiri speakers, excluding those who communicate in Maay or other minority languages. Although telehealth platforms have expanded access to medical consultations in rural areas, their lack of multilingual support limits their inclusivity. Health information disseminated through text messages or audio recordings is typically delivered only in Maxaa Tiri, leaving minority language speakers unable to benefit fully from these services [4]. Despite these limitations, digital health platforms present significant opportunities to bridge language gaps. Incorporating multilingual support tools into the existing telehealth systems can ensure broader accessibility [13].

AI‐driven translation technologies can facilitate real‐time communication between patients and providers who speak different languages [15]. Voice‐based interfaces could further enhance accessibility for low‐literacy populations by allowing users to interact with platforms that use spoken language rather than written text [16]. By integrating these innovations into digital health platforms, the Federal Republic of Somalia can create more inclusive systems that address the needs of all linguistic groups, while expanding access to quality care across the country. To effectively bridge linguistic disparities, the Federal Republic of Somalia can adopt innovative solutions, including mobile health applications integrated with multilingual chatbots and digital interpreter training platforms. These digital innovations can help tailor healthcare delivery to the specific linguistic needs of minority communities, enhancing inclusivity and access.

## 2 Policy Gaps and Recommendations

The Federal Republic of Somalia's Provisional Constitution recognizes both Maxaa Tiri and Maay as official dialects but lacks enforceable frameworks mandating language inclusivity in critical sectors such as healthcare [17]. This policy gap disproportionately affects minority language speakers, who face systemic barriers when accessing services designed primarily for Maxaa Tiri speakers [1, 2, 4]. Several countries provide transferable lessons. South Africa's National Department of Health Language Policy (2023) obliges every facility to communicate in all 11 official languages and South African Sign Language, creating an accountability mechanism for compliance [18]. Tanzania's 2024 Community Health Worker curriculum includes compulsory training in basic interpretation and sign language, extending language support to the frontline of care [19]. India's Tele‐MANAS national mental health helpline, launched in 2022, already handles thousands of calls daily in 20 Indian languages, showing how multilingual telemedicine can reach linguistically diverse rural populations [20]. Nepal's National Health Communication Policy requires that all nationwide health messages be prepared in multiple local languages before dissemination, ensuring that no community is excluded from receiving vital information [21].

These examples demonstrate successful strategies that the Federal Republic of Somalia could adapt; the Federal Republic of Somalia must develop a national policy on language inclusivity that is aligned with UHC goals. Public healthcare facilities are required to provide services in both Maxaa Tiri and Maay, with additional support for minority languages, where necessary. Translating consent forms, medical records, and public health materials into multiple dialects ensures broader accessibility. Countries like Canada implement federally funded interpreter programs supporting many languages, significantly reducing language‐related healthcare disparities [22]. Similarly, India effectively utilizes multilingual telemedicine services and locally tailored mobile health applications to expand healthcare access in linguistically diverse rural areas [23]. Although specific comparative data for the Federal Republic of Somalia is unavailable, these examples demonstrate successful strategies the Federal Republic of Somalia could adapt. A national program should also be established to train interpreters fluent in minority languages to bridge communication gaps within hospitals and clinics [24]. Adequate funding must be allocated to translation services, interpreter training programs, and multilingual digital health platforms that incorporate AI‐driven translation technologies for real‐time communication between patients and providers [15]. Finally, monitoring mechanisms should be implemented to ensure compliance with these policies while incorporating feedback from minority communities into future revisions.

## 3 The Path Toward UHC

Achieving UHC in the Federal Republic of Somalia requires addressing inequities that prevent minority language speakers from accessing health care. Language inclusivity ensures that all individuals, regardless of their linguistic background, receive quality care [25]. Excluding minority language speakers undermines equity and accessibility, leaving marginalized communities vulnerable to poor health outcomes. Effective communication is essential for an accurate diagnosis, informed consent, and treatment adherence [3]. Linguistic barriers disproportionately affect vulnerable populations and limit public health initiatives such as vaccination campaigns when information is not provided in native dialects [2]. Addressing these gaps is central to equitable health care. To integrate language inclusivity into UHC, the Federal Republic of Somalia must prioritize marginalized communities by mandating multilingual services in healthcare facilities and allocating resources for interpreter training and translation services [24]. Digital health innovations such as multilingual telehealth platforms and mobile apps with audio‐visual content in multiple dialects should be leveraged to expand access [13]. Community‐based approaches, including training health workers fluent in minority dialects, can enhance primary care delivery and build trust between healthcare providers and local communities [24]. International collaboration with organizations such as the WHO can provide technical expertise and funding for inclusive initiatives.

## 4 Conclusion

Addressing language barriers is essential to achieve equitable access to healthcare in the Federal Republic of Somalia. Maxaa Tiri's dominance in healthcare marginalizes speakers of Maay and other minority languages, limiting their ability to access services, understand medical procedures, and participate in public health initiatives. These barriers perpetuate systemic inequities, resulting in poorer health outcomes among vulnerable populations, particularly in rural and linguistically diverse regions. Solutions must focus on integrating linguistic inclusiveness into national healthcare frameworks. This includes mandating multilingual services, training interpreters, and leveraging digital tools, such as AI‐powered translation platforms and multilingual telehealth applications. Community‐based approaches, such as training health workers fluent in minority dialects, can further bridge communication gaps while fostering trust between providers and marginalized communities. The experiences of countries such as South Africa, Tanzania, India, Nepal, and Canada offer valuable lessons in multilingual health policy, digital inclusion, and interpreter services. These global models illustrate that targeted investments and policy reforms can overcome linguistic exclusion in healthcare systems. The Federal Republic of Somalia can adapt these lessons to its unique socioeconomic context by prioritizing grassroots solutions while gradually building institutional capacity. UHC requires ensuring that no one is excluded because of linguistic barriers. By prioritizing language diversity within its healthcare system, the Federal Republic of Somalia can advance equity, improve health outcomes, and create an inclusive future in which all citizens, both urban and rural, have access to quality care, regardless of their linguistic background.

## Author Contributions

**Najib Isse Dirie:** conceptualization (lead), writing – review and editing (equal). **Mohamed Mustaf Ahmed:** data curation (lead), formal analysis (lead), writing – original draft (lead), writing – review and editing (equal).

## Ethics Statement

The authors have nothing to report.

## Consent

The authors have nothing to report.

## Conflicts of Interest

The authors declare no conflicts of interest.

## Data Availability

The authors have nothing to report.
